# Comprehensive safety evaluation of DW2009, a complex of *Lactiplantibacillus* C29 and fermented soybean powder

**DOI:** 10.1016/j.toxrep.2026.102299

**Published:** 2026-06-24

**Authors:** Hyunju Kim, Dong-Hyun Kim, Ji-Su Baek, Daeyeon Won

**Affiliations:** a DONGWHA Pharm Research Institute, Giheung-gu, Yongin-si 17084, Korea; b College of Pharmacy, Kyung Hee University, Dongdaemun-gu, Seoul 02447, Korea

**Keywords:** DW2009, *Lactiplantibacillus plantarum* C29, Probiotics, Fermented soybean powder, Safety evaluation

## Abstract

Probiotics with potential health benefits are increasingly incorporated into a wide range of functional foods. However, the safety profiles of probiotics can vary depending on the strain, necessitating a comprehensive safety assessment prior to human use. In this study, the safety profile of a complex of *Lactiplantibacillus plantarum* C29 and fermented soybean powder (DW2009) was evaluated through a battery of *in vitro* and *in vivo* toxicological assessments. *L. plantarum* C29 demonstrated susceptibility to all tested antibiotics, thereby minimizing the risk of horizontal gene transfer. Safety tests confirmed the absence of hemolytic activity, virulence factors, toxin production, biogenic amine production, and mucin degradation. The results of a 90-day oral toxicity study with repeated doses established no observable adverse effect for DW2009 at 3000 mg/kg body weight/day. Additionally, all genotoxicity assays yielded negative results, indicating no mutagenic potential. Taken together, these findings support a favorable safety profile of DW2009 for application in functional foods.

## Highlights

• *Lactiplantibacillus plantarum* C29 lacks antibiotic resistance and virulence factors.
• In vitro assays confirmed no hemolytic, cytotoxic, or toxin-producing activity in C29.
• Sub-chronic toxicity study showed no treatment-related adverse effects.
• The NOAEL for DW2009 was determined to be 3000 mg/kg/day.
• Genotoxicity tests confirmed the non-mutagenic potential of DW2009.

## Graphical Abstract

**Figure:**
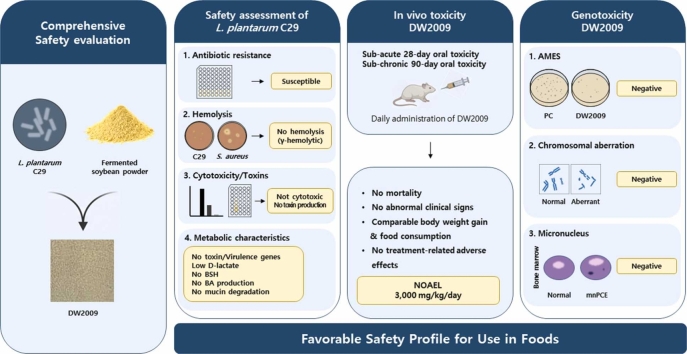

## 1 Introduction

Probiotics are live microorganisms that confer health benefits when administered in adequate amounts and are widely applied in functional foods. Lactic acid bacteria (LAB), particularly *Lactobacillus*, are probiotic microorganisms with established health-promoting properties through modulation of intestinal microbiota composition and immune function [1], [2]. Many LAB species have been granted qualified presumption of safety status by the European Food Safety Authority (EFSA) and are generally recognized as safe by the U.S. Food and Drug Administration [3], [4]. Although the safety profiles of individual strains of LAB are well documented, accumulating evidence of strain-dependent antibiotic resistance patterns, metabolic activities, host interactions, and potential toxicological effects necessitates comprehensive evaluations prior to use in commercial food production [5], [6].

*Lactiplantibacillus plantarum* is commonly isolated from dairy products and fermented vegetables. Due to an extensive history of human consumption, *L*. *plantarum* is generally considered safe. Numerous studies have reported that *L. plantarum* is tolerated well by both healthy individuals and patients with gastrointestinal conditions [7], [8]. Although *L. plantarum* is typically regarded as safe, recent studies have identified strain-specific concerns, including antibiotic resistance acquisition and potential virulence factor expression in certain strains [9], [10], [11], [12], thereby underscoring the importance of strain-level safety characterization.

DW2009 is a probiotic complex comprising *L. plantarum* C29, originally isolated from Kimchi (a traditional Korean fermented cabbage) and soybean powder fermented by *L. plantarum* C29. *L. plantarum* C29 was reported to exhibit the most pronounced cognition-enhancing effect among 30 strains isolated from Kimchi [13] and efficacious in multiple animal models [14], [15]. Furthermore, fermentation of soybean powder with *L. plantarum* C29 augments its cognitive benefits [16], providing a sound rationale for the synergistic combination of *L. plantarum* C29 and fermented soybean powder. Recent human and murine studies demonstrated that DW2009 improved cognitive function through normalization of the gut microbiota and reduction of neuroinflammation. In a clinical trial, participants with mild cognitive impairment who received DW2009 for 12 weeks showed greater improvement in combined cognitive functions, especially in attention/prefrontal function in computerized neurocognitive function tests [17], [18], [19], [20]. Nevertheless, the safety profile of this complex requires further characterization.

Although soybean (Glycine max) is widely utilized as a conventional food ingredient with an established safety profile, its fermentation using *L. plantarum* C29 represents a novel ingredient. Notably, this specific fermented soybean powder has no history of human consumption until the development of DW2009. The fermentation process involves enzymatic biotransformation that can alter the chemical composition, potentially yielding new metabolites that require toxicological characterization. Furthermore, regulatory authorities typically do not grant the status of substantial equivalence to fermented products or complexes relative to their raw materials. Consequently, comprehensive toxicological assessments are mandatory to evaluate the safety and potential risks associated with DW2009.

Therefore, the aim of the present study was to evaluate the safety profile of DW2009 through toxicological, genomic, and metabolic assessments using *in vitro* and *in vivo* assays. Also, *in vitro* characterization was conducted to verify the strain-level safety of *L. plantarum* C29. Subsequently, the safety of DW2009 was assessed by 28- and 90-day oral toxicity studies with repeated doses, complemented by genotoxicity screening. Collectively, the results provide experimental evidence supporting the safe application of the DW2009 complex in food and dietary supplements.

## 2 Materials and methods

### 2.1 Preparation of *L. plantarum* C29 and DW2009

For probiotic safety studies, *L. plantarum* C29 (KCCM 11291 P) was cultured in De Man, Rogosa, and Sharpe (MRS) medium unless stated otherwise. DW2009 used for the *in vivo* toxicology and genotoxicity studies was manufactured by CheBiGen Inc. (Jeonju, Korea).

### 2.2 *L. plantarum* C29 toxicity study

#### 2.2.1 Minimum inhibitory concentration (MIC) of antibiotics

The MIC of *L*. *plantarum* C29 against seven clinically relevant antibiotics (ampicillin, gentamycin, kanamycin, erythromycin, clindamycin, tetracycline, and chloramphenicol) was determined using the broth microdilution method, as described in the 2012 EFSA guidelines [21], to evaluate the antibiotic resistance profile. A 96-well plate was incubated at 35°C for 16–20 h and the optical density at 600 nm was measured using an Epoch−2 Microplate Spectrophotometer (BioTek Instruments, Winooski, VT, USA). In parallel, antibiotic microbial resistance genes encoded by the *L*. *plantarum* C29 genome were identified using the Resistance Gene Identifier 6.0.5 tool in the Comprehensive Antibiotic Resistance Database 4.0.1 (https://card.mcmaster.ca/) using default parameters and the ResFinder 4.7.2 tool (https://pypi.org/project/resfinder/) with default parameters, identity of 80%, and minimum length threshold of 60% [22].

#### 2.2.2 Hemolysis

To assess hemolytic activity, cultured *L. plantarum* C29 cells were streaked on agar plates supplemented with 5% (w/v) defibrinated sheep blood and incubated at 37°C for 48 h. *Staphylococcus aureus* subsp*. aureus* (ATCC6538) was used as a positive control for β-hemolysis [23], [24]. Hemolytic activity was determined based on the appearance of hemolytic zones around the colonies, which indicate the breakdown of red blood cells (green-hued halo, α-hemolysis; clear halo, β-hemolysis; no color change, γ-hemolysis [nonhemolytic]).

#### 2.2.3 Cytotoxicity and toxin production

The cytotoxicity of *L*. *plantarum* C29 was assessed using a commercial lactate dehydrogenase (LDH) assay kit (Dojindo Laboratories, Kumamoto, Japan) in accordance with the manufacturer’s instructions. Briefly, 10^7^∼10^9^ colony-forming units (CFU) of *L. plantarum* C29 or *Escherichia coli (E. coli)* in respective serum-free cell culture medium were added to a monolayer of HT29 cells in the wells of a 96-well plate and incubated for 24 h at 37°C under an atmosphere of 5% CO_2_/95% air. Vehicle-treated cells served as a negative control, and lysis buffer–treated cells served as a positive control. After incubation, the supernatants were collected and 50-μL aliquots were transferred to the wells of a new 96-well plate. Following the addition of the LDH assay mixture, the plate was incubated at room temperature for 30 min. Afterward, the absorbance at 450 nm was measured with an Epoch−2 Microplate Spectrophotometer.

Enterotoxin production was assessed using the *Bacillus cereus* Enterotoxin Reversed Passive Latex Agglutination Kit (BCET-RPLA) (Oxoid Ltd., Basingstoke, Hampshire, UK) [25]. *L. plantarum* C29 cells were cultured in brain-heart infusion broth at 37°C with shaking at 150 rpm for 16–18 h. Afterward, the cells were harvested and the supernatants were tested in accordance with the manufacturer’s protocol.

Endotoxin levels were determined using the *Limulus* amebocyte lysate Chromogenic Endotoxin Quantitation Kit (Thermo Fisher Scientific, Waltham, MA, USA) [26]. *L. plantarum* C29 cells were incubated at 37°C for 20–24 h. Following centrifugation, the resulting bacterial pellets were resuspended in endotoxin-free phosphate-buffered saline (PBS) and processed in accordance with the manufacturer’s instructions. Afterward, the absorbance at 450 nm was measured.

#### 2.2.4 Determination of virulence factors, toxins, and undesirable genes

Whole-genome sequencing was conducted by CJ Bioscience Inc. (Seoul, Korea). Briefly, the genome of *L. plantarum* C29 was constructed *de novo* using a commercial dataset (Pacific Biosciences Inc., Menlo Park, CA, USA). The sequencing data were assembled using Flye 2.9.2 software in accordance with the microbial analysis protocol. Resulting contigs from the PacBio sequencing data were circularized using the Circlator 1.4.0 tool (https://sanger-pathogens.github.io/circlator/). The genes encoding virulence factors and toxins of *L. plantarum* C29 were analyzed using the software tools ToxFinder 1.0, Virulence Finder 2.0, and PathogenFinder 1.1 [22]. The whole-genome sequence of *L. plantarum* C29 was uploaded, with the sequencing platform set to Assembled Genome/Contig and the remaining parameters maintained at default settings.

#### 2.2.5 D-lactate quantification

In order to quantify the content of D-lactate, *L. plantarum* C29 was incubated in MRS broth at 37°C for 24 h. The supernatants were analyzed using a D-lactate assay kit (Abcam Inc., Waltham, MA, USA). The absorbance was measured at 450 nm. Supernatants of vehicle-treated cells served as a negative control.

#### 2.2.6 Bile salt deconjugation

Bile salt hydrolase (BSH) activity was measured using sodium glycocholate and sodium taurocholate, which are conjugated forms of cholic acid with glycine and taurine, as previously reported [27]. For the bile salt deconjugation assay, 100 μL of purified protein samples or *L. plantarum* C29 cells were mixed with 1.8 mL of 0.1 M sodium-phosphate buffer (pH 6.0) and 100 μL of either 20 mM sodium glycocholate or 20 mM sodium taurocholate. Following incubation at 37°C for 30 min, 500 μL of the reaction mixture were mixed with 500 μL of 15% (w/v) trichloroacetic acid to terminate the reaction. To estimate the concentrations of the liberated amino acids (glycine or taurine) from the conjugated bile acids, 20 μL of the above mixture were diluted with 80 μL of distilled water and mixed with 1.9 mL of ninhydrin reagent (0.5 mL 1% ninhydrin in 0.5 M sodium citrate buffer pH 5.5, 1.2 mL 30% glycerol, 0.2 mL 0.5 M sodium citrate buffer pH 5.5). The reaction mixture was boiled for 14 min for coloration. Absorbance at 570 nm was measured using an Epoch−2 Microplate Spectrophotometer. The amino acid concentration was estimated against a standard curve of either glycine or taurine based on the conjugated bile salts used. BSH activity was further confirmed using MRS agar supplemented with 0.5% (w/v) taurodeoxycholic acid. Plates were cultured at 37°C for 48 h. The formation of precipitation zones around colonies was classified as exhibiting BSH activity.

#### 2.2.7 Biogenic amine (BA) production

The BA-producing ability of *L. plantarum* C29 was assessed as previously described [28]. Briefly, the test strain was cultured on Moeller decarboxylase broth base agar supplemented with specific amino acids (1% (w/v) each of arginine, histidine, lysine, ornithine, and phenylalanine) at 37°C for 16–24 h. A color change from yellow to purple of the agar surrounding the colonies indicated positive decarboxylase activity. *Lactobacillus rhamnosus* GG and *Listeria monocytogenes* (KACC10764) were used as negative controls, and *Yersinia enterocolitica* subsp. *enterocolitica* (KACC15320) as a positive control.

#### 2.2.8 Mucin degradation

Mucin degradation was assessed using a previously established protocol with minor modifications [29]. Briefly, a Gifu anaerobic medium agar plate supplemented with 0.5% (w/v) hog gastric mucin with or without the addition of 3% (w/v) glucose was inoculated with *L. plantarum* C29, the negative controls *L. rhamnosus* GG and *L. monocytogenes* (KACC10764), and the positive control *Y. enterocolitica* (KACC 15320), incubated at 37°C for 72 h, and then stained with 0.1% amido black in 3.5 M acetic acid for 30 min. A halo zone surrounding the colonies was observed after washing with 1.2 M acetic acid. The absence of the halo zone was interpreted as the lack of mucinolytic activity.

### 2.3 DW2009 toxicity study

#### 2.3.1 Animals

Five-week-old Sprague Dawley rats of both sexes (100–150 g) were obtained from OrientBio Inc. (Seongnam, Korea). The animals were housed under standard conditions at room temperature (22°C ± 3°C) under a 12-h light:dark cycle with *ad libitum* access to food and water. Prior to experimentation, the rats were acclimated for one week. All *in vivo* studies were approved by the Institutional Animal Care and Use Committee of Biotoxtech Co., Ltd. (IACUC approval numbers: 180082 for the 28-day study and 200395 for the 90-day study).

#### 2.3.2 Subacute oral toxicity study (28 days)

Male and female Sprague Dawley rats (age, 6 weeks; n = 5/sex/group) were randomly allocated to four groups to achieve even distribution of mean body weight of each group. DW2009 was administered at doses of 750, 1500, and 3000 mg/kg body weight/day. The test material was prepared daily by dissolution in water for oral gavage at 12 mL/kg body weight once daily for 28 consecutive days. Control animals received only the vehicle. Subacute repeated-dose oral toxicity study was performed following Organization for Economic Co-operation and Development (OECD) Test Guideline 407 (“Repeated Dose 28-day Oral Toxicity Study in Rodents”).

#### 2.3.3 Subchronic oral toxicity study (90 days)

Male and female Sprague Dawley rats (age, 6 weeks; n = 10/sex/group) were randomly assigned to four groups to achieve even distribution of mean body weight of each group and administered either a vehicle or DW2009 via oral gavage once daily for 90 consecutive days. DW2009 at doses of 750, 1500, and 3000 mg/kg body weight/day was dissolved in water daily and administered by oral gavage at 12 mL/kg body weight. This study was conducted in accordance with the Principles of Good Laboratory Practice of the OECD (ENV/MC/CHEM(98)17) and Test Guideline 408 (“Repeated Dose 90-day Oral Toxicity Study in Rodents”).

#### 2.3.4 Evaluation of parameters

##### 2.3.4.1 Clinical observations

The rats were monitored once daily for clinical signs and twice daily for mortality and moribundity. Clinical observations included skin, fur, eyes, mucous membranes, occurrence of secretion and excretion, autonomic activity (e.g., lacrimation, piloerection, pupil size, abnormal respiration), clonic or tonic movements, stereotypies (e.g., excessive grooming, repetitive circling), and abnormal behavior (e.g., self-mutilation, walking backward), in accordance with OECD Test Guidelines 407 and 408. Body weight and food consumption were recorded weekly.

##### 2.3.4.2 Hematological, biochemical, and urinary analyses

All animals were fasted overnight for approximately 18 h prior to necropsy. Before blood sample collection, the animals were anesthetized with isoflurane, and blood samples were collected from the abdominal aorta. For hematological analysis, blood samples were collected into vacutainers containing ethylenediaminetetraacetic acid. Blood parameters were analyzed using an autoanalyzer (ADVIA 2120i, Siemens AG, Munich, Germany or XN-V, Sysmex Corporation, Kobe, Japan). For the coagulation test, blood samples were collected into tubes containing 3.2% sodium citrate and centrifuged at 3000 rpm for 10 min to separate the plasma. The prothrombin time and activated partial thromboplastin time were measured using a Coapresta 2000 (Sekisui Chemical, Tokyo, Japan).

For clinical chemical analysis, blood samples were collected from the abdominal aorta and centrifuged at 2000 × *g* for 10 min to obtain serum. The parameters were analyzed using a 7180 clinical analyzer (Hitachi, Ltd., Tokyo, Japan) and EasyLyte (Medica Corporation, Bedford, MA, USA).

The rats were housed in separate metabolic cages for urine collection prior to euthanasia. The samples were observed for color and turbidity. The protein, ketone body, bilirubin, and occult blood contents and the pH value were determined using Combur-Test® strips (Roche Diagnostics GmbH, Mannheim, Germany) or the cobas® u 411 urine analyzer (Roche Diagnostics GmbH). Specific gravity was measured using a VET 360 Refractometer (Ametek Reichert Technologies, Depew, NY, USA).

### 2.4 Genotoxicity

#### 2.4.1 Bacterial reverse mutation test (Ames test)

The Ames test was conducted to assess the mutagenicity of DW2009 in accordance with OECD Test Guideline 471 (“Bacterial Reverse Mutation Test”) using *Salmonella typhimurium* histidine-auxotrophic strains TA98, TA100, TA1535, and TA1537, along with *E. coli* tryptophan-auxotrophic strain WP2uvrA (pKM101) (Molecular Toxicology Inc., Boone, NC, USA). DW2009 was suspended in normal saline, and the S9 metabolic activation system was prepared using S9 mix and cofactor A (Oriental Yeast Co., Ltd., Tokyo, Japan) before start of experiment.

Briefly, 100 μL of DW2009 solution at concentrations of 5.0, 2.5, 1.25, 0.625, and 0.3125 mg/plate, strain-specific positive and negative controls were placed in the respective tubes. Following the addition of 500 μL of PBS (−S9 group) or 500 μL of of S9 mix (+S9 group) and 100 μL of preincubated bacterial suspension, the mixtures were incubated at 37°C and 90 rpm for 20 min. Then, 2 mL of top agar solution containing 45 μM Histidine (for *S. typhimurium*) or 45 μM tryptophan (for *E. coli*) were added to each strain and poured on minimal glucose agar plates. After solidification, the plates were incubated at 37°C for 48 h, followed by enumeration of revertant colonies. PBS was used as a negative control, whereas known mutagens (listed in Table 1) were included as positive controls. A sample was considered mutagenic if (i) there was a twofold or greater increase in the number of revertant colonies relative to the negative control in one or more strains and (ii) the increase was dose-dependent or reproducible.

**Table 1:** Positive controls for the Ames test.

**TA strain**	**Without S9 activation**	**μg/plate**	**With S9 activation**	**μg/plate**
TA98	2-Nitrofluorene	5.0	2-Aminoanthracene	1.0
TA100	Sodium azide	1.5	2-Aminoanthracene	2.0
TA1535	Sodium azide	1.5	2-Aminoanthracene	3.0
TA1537	9-Aminoacridine	80.0	2-Aminoanthracene	3.0
WP2uvrA (pKM101)	4-Nitroquinoline N-oxide	0.1	2-Aminoanthracene	2.0

#### 2.4.2 *In vitro* chromosomal aberration test

The chromosomal aberration test was performed in accordance with OECD Test Guideline 473 (“*In Vitro* Mammalian Chromosomal Aberration Test”). Chinese hamster lung CHL/IU cells were cultured in Eagle’s minimum essential medium (Lonza Walkersville Inc., Walkersville, MD, USA) supplemented with 10% fetal bovine serum (Gibco®, Thermo Fisher Scientific, Waltham, MA, USA). Cells (2.5 ×10^5^) were seeded in a 60-mm dish and incubated at 37°C for 24 h. A cytotoxicity assay was conducted with test substance concentrations of 19.5, 39.1, 78.1, 156, 313, 625, 1250, 2500, and 5000 μg/mL. Based on the results of the cytotoxicity assay, the exposure concentrations for short-term (6 h) treatment with S9 were 625, 1250, 2500, and 5000 μg/mL; for short-term treatment without S9 were 1250, 2500, and 5000 μg/mL; and for long-term (24 h) treatment without S9 metabolic activation were 40.6, 81.3, 163, 325, and 650 μg/mL. DW2009 was freshly prepared in culture medium, and cells were exposed to DW2009 either in the presence or absence of an S9 mix (Oriental Yeast Co., Ltd.) at 37°C.

For short-term treatments, the cells were treated with the test substance for 6 h, washed with DPBS, resuspended in complete cell culture medium, and incubated for 18 h. For long-term treatment, cells were treated with the test substance for 24 h. Mitomycin C (10 μg/mL; Sigma-Aldrich Corporation, St. Louis, MO, USA) and benzo[*a*]pyrene (2 mg/mL; Sigma-Aldrich Corporation) were used as positive controls in the absence and presence of S9 mix, respectively. Then, 300 metaphases per dose were observed under a microscope (600 × magnification, BX51, Olympus Corporation, Tokyo, Japan) and chromosomal abnormalities were categorized as structural, numerical, or other. Cells exhibiting one or more gaps or breaks were considered fragmented, while those exhibiting one or more aberrations were considered aberrant. The types were recorded individually.

#### 2.4.3 Micronucleus test

Male Institute of Cancer Research mice (7 weeks old; n = 25) were obtained from OrientBio Inc. (Seongnam, Korea). The animals were housed in groups of five per cage under controlled conditions with a 12-h light:dark cycle, a constant temperature of 22°C ± 3°C, and relative humidity of 40%–70%, with *ad libitum* access to food and water. Prior to experimentation, the mice were acclimated for 1 week. All experimental procedures were approved by the Institutional Animal Care and Use Committee of Biotoxtech (IACUC approval no. 180189) and conducted in accordance with OECD Test Guideline 474 (“Mammalian Erythrocyte Micronucleus Test”).

Mice were randomly assigned to five groups. Mice received oral gavage with DW2009 at 1250 (low dose), 2500 (middle dose), or 5000 (high dose) mg/kg body weight/day at 24-h intervals. Mitomycin C (Sigma-Aldrich Corporation) at 2 mg/kg was administered only once via intraperitoneal injection as a positive control, whereas the vehicle was used as a negative control. All animals were sacrificed by cervical vertebral dislocation 24 h after the final injection. Immediately following sacrifice, bone marrow cells were harvested from the femur and centrifuged at 1000 rpm for 5 min at 4°C. After discarding most of the supernatant, the remaining supernatant was mixed well with the precipitate. Cells were then spread on a glass slide, air-dried, fixed with absolute methanol, and stained with 3% Giemsa solution for 30 min. Micronucleus formation was assessed under a microscope (BX51, Olympus Corporation) at 600 × magnification. For each mouse, 4000 polychromatic erythrocytes (PCEs) were examined, and the ratio of micronucleated PCEs to PCEs was calculated.

### 2.5 Statistical analysis

Statistical analyses for the cytotoxicity and D-lactate production experiments were performed using Prism 10 software (GraphPad Software, LLC, San Diego, CA, USA). Differences between multiple groups were compared by one-way analysis of variance (ANOVA), followed by Tukey’s multiple comparisons test.

Statistical analyses for the *in vivo* toxicity and genotoxicity tests were conducted with SAS software (version 9.3; SAS institute Inc., Cary, NC, USA). Data were assessed for homogeneity of variance using the Bartlett test. For data with homogeneous variance, one-way ANOVA followed by Dunnett’s *t*-test was generally used to evaluate the statistical significance of the results. If the variance was not homogenous, the Kruskal–Wallis test was employed. For the micronucleus test, the incidence of micronucleated bone marrow polychromatic erythrocytes (mnPCEs) was compared between the negative control group and each treatment group or the positive control using the Mann–Whitney test. For the chromosomal aberration test, the incidence of aberrations was analyzed using Fisher’s exact test to compare the negative control group with each treatment group and the positive control group. Two-way ANOVA for hematological, biochemical, and urinary parameters was performed using Prism 10 software (GraphPad Software, LLC, San Diego, CA, USA). For all analyses, statistical significance was defined as a probability (*p*) value < 0.05.

## 3 Results

### 3.1 *L. plantarum* C29 toxicological study

#### 3.1.1 Antibiotic resistance

Antimicrobial susceptibility testing of *L. plantarum* C29 was conducted by determining the MICs for a panel of seven clinically relevant antibiotics (ampicillin, gentamycin, kanamycin, erythromycin, clindamycin, tetracycline, and chloramphenicol). The measured MIC values were below the cut-off values for *L. plantarum* as defined by the EFSA guidelines, indicating susceptibility to the tested antibiotics. Genome-based analysis using the Comprehensive Antibiotic Resistance Database identified one putative vancomycin resistance-associated gene (*vanY*) encoded by the chromosome of *L. plantarum* C29. The *vanY* gene is chromosomally encoded and nontransferable, thereby possessing minimal potential for horizontal gene transfer to pathogenic bacteria [21]. Additionally, no acquired antibiotic microbial resistance gene or chromosomal mutation related to horizontal gene transfer was identified with the ResFinder 4.7.2 tool (Table 2).

**Table 2:** Antibiotic susceptibility profile of *L. plantarum* C29.

**Antibiotic**	**Cut-off value (mg/L)**^a^	**MIC**^b^	**Susceptibility**	**Resistance gene**
Ampicillin	2	1	susceptible	not detected
Gentamycin	16	1	susceptible	not detected
Kanamycin	64	32	susceptible	not detected
Erythromycin	1	0.125	susceptible	not detected
Clindamycin	2	0.125	susceptible	not detected
Tetracycline	32	16	susceptible	not detected
Chloramphenicol	8	4	susceptible	not detected

a Cut-off value based on the EFSA guidelines

b Minimum inhibitory concentration

#### 3.1.2 Hemolysis

**Fig. 1:**
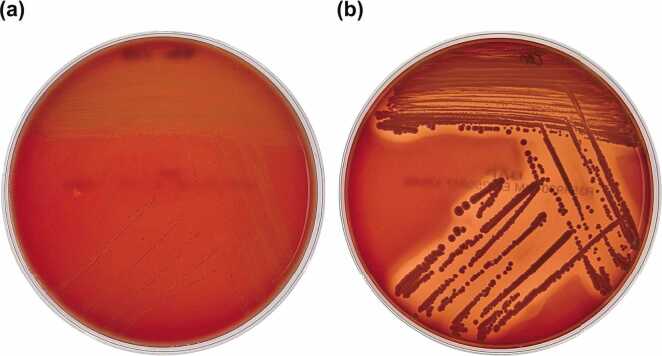
Hemolysis of *L. plantarum* C29. (**a**) *L. plantarum* C29; (**b**) *S. aureus*.

The hemolytic activity of *L. plantarum* C29 was evaluated using an agar plate-based assay. Following incubation, there were no zones of hemolysis (clear or greenish) surrounding the *L*. *plantarum* C29 colonies. In contrast, *S. aureus* subsp*. aureus* induced distinct clear zones indicative of β-hemolysis (Fig. 1). These results confirmed that *L. plantarum* C29 is safe (γ-hemolytic).

#### 3.1.3 Cytotoxicity and toxin production

**Fig. 2:**
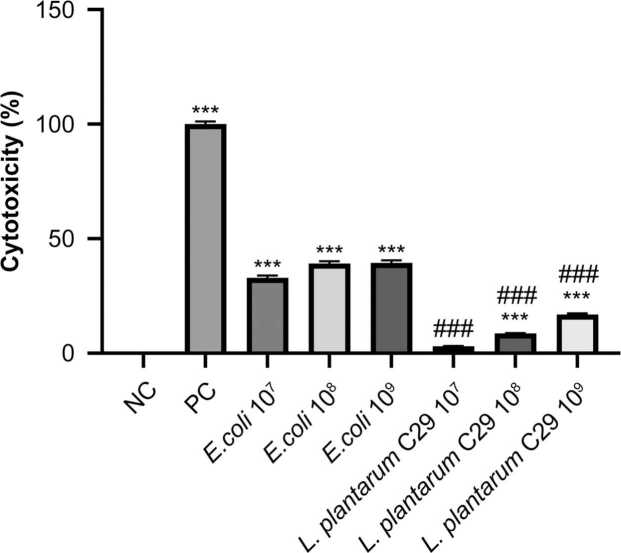
Cytotoxic effect of *L. plantarum* C29 on HT29 cells. The data are presented as the mean ± SE. Significantly different from the negative control as determined by ANOVA followed by Tukey’s multiple comparisons test: ***p < 0.001. Significantly different from *E. coli* at the same dose (cfu) as determined by ANOVA followed by Tukey’s multiple comparisons test: ###p < 0.001. NC = negative control, PC = positive control.

The cytotoxicity of *L. plantarum* C29 in HT29 cells was evaluated using the LDH assay. Treatment of HT29 cells with pathogenic *E. coli* resulted in cytotoxicity (10^7^ CFU, 32.9 ± 0.98%; 10^8^ CFU, 39.2 ± 0.95%; 10^9^ CFU, 39.4 ± 1.07%). In contrast, treatment with *L. plantarum* C29 exhibited limited cytotoxicity (10^7^ CFU, 3.1 ± 0.18%; 10^8^ CFU, 8.7 ± 0.14%; 10^9^ CFU, 16.9 ± 0.53%) (Fig. 2).

Since *B. cereus* in food produces several enterotoxins that cause diarrheal syndrome [30], the presence of *B. cereus* enterotoxin in the culture medium of *L. plantarum* C29 was assessed using a *B*. *cereus* Enterotoxin Reversed Passive Latex Agglutination Kit. No agglutination of sensitized latex particles was observed, whereas the positive control exhibited agglutination (Supplementary Table 1).

Endotoxin levels in the culture medium of *L. plantarum* C29 were measured using a chromogenic assay. The endotoxin concentration was 0.041 ± 0.0003 EU/mL, which was close to the minimum limit of quantitation of the assay. These results show that *L. plantarum* C29 is not cytotoxic and does not produce toxins.

#### 3.1.4 Determination of toxin genes and virulence factors

Screening of the *L. plantarum* C29 genome using ToxFinder found no toxin-associated genes, while screening with VirulenceFinder v2.0 identified no virulence-associated genes. Furthermore, PathogenFinder predicted a low probability of the strain being a human pathogen (score = 0.206).

#### 3.1.5 Metabolic characteristics

**Fig. 3:**
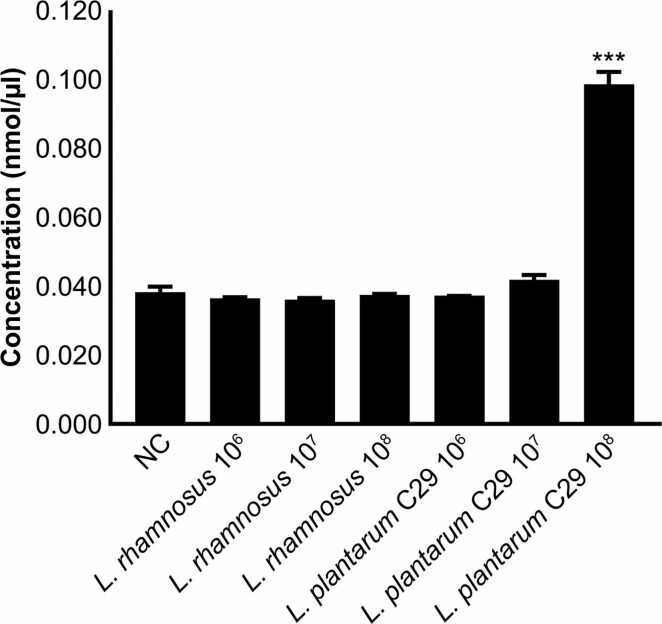
D-lactate production by *L. plantarum* C29. The data are presented as the mean ± SE.Significantly different from negative control by ANOVA with Tukey’s multiple comparisons test: ***p < 0.001. NC = negative control.

After 24 h of incubation, D-lactate levels in *L. plantarum* C29 at 10⁶ and 10⁷ CFU were comparable to those of the negative control (0.037 ± 0.0003, 0.042 ± 0.002 vs. 0.038 ± 0.002 nmol/μL). A higher D-lactate level was observed only at the highest tested dose (10⁸ CFU. 0.099 ± 0.0003 nmol/μL), which was statistically different from the negative control (Fig. 3).

**Fig. 4:**
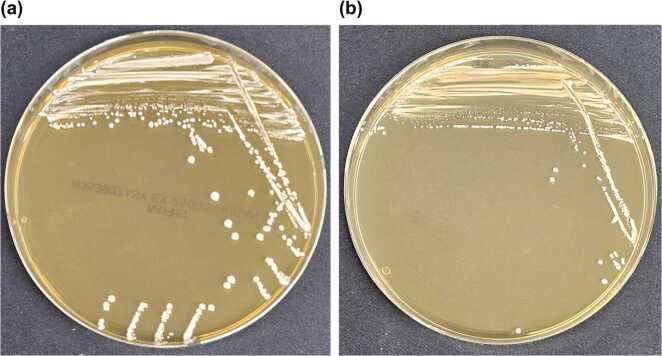
Bile salt deconjugation of *L. plantarum* C29. (**a**) *L. plantarum* C29 incubated on MRS agar plate; (**b**) *L. plantarum* C29 incubated on MRS agar plate with 0.5% TDCA.

BSH activity was measured by assessing hydrolysis of conjugated bile salts. The amounts of glycine and taurine deconjugated from bile salts by *L. plantarum* C29 were comparable to those of the negative control, with no significant difference observed under the tested conditions (Table 3). In the agar plate-based assay, no precipitation halo around the *L. plantarum* C29 colonies was observed on the MRS agar plate with or without 0.5% taurodeoxycholic acid (Fig. 4). These results demonstrate that *L*. *plantarum* C29 lacks BSH activity.

**Table 3:** Deconjugated bile salts by *L. plantarum* C29 cells and lysates.

**Sample**	**Concentration (μM)**	
	**Glycine**	**Taurine**
NC	1.12 ± 0.055	4.45 ± 0.246
C29	0.99 ± 0.004	4.24 ± 0.085
C29 lysate	1.07 ± 0.016	4.57 ± 0.057

BAs are usually found in fermented foods, such as cheese, wine, and fermented vegetables. However, excessive intake of BAs can cause nausea, gastrointestinal problems, hypertension, and cancer [31]. On BA indicator agar, *Y. enterocolitica* formed large colonies accompanied by a purple color change to the surrounding medium. In contrast, no color change was observed in the agar surrounding colonies of *L. plantarum* C29, *L. rhamnosus* GG, or *L. monocytogenes*, indicating the absence of BA production.

There was no halo zone surrounding colonies of *L. plantarum* C29, *L. rhamnosus* GG, and *L. monocytogenes* on the agar plate containing 0.3% mucin as the sole carbon source. In contrast, a halo zone was observed around *the Y. enterocolitica* colonies. These results demonstrate that *L. plantarum* C29 does not exhibit BA-producing and mucin-degrading activities (Table 4).

**Table 4:** BA production and mucin degradation activity of *L. plantarum* C29.

	***L. rhamnosus*** **GG**	***L. monocytogenes***	***Y. enterocolitica***	***L. plantarum*****C29**
BA production	Negative	Negative	Positive	Negative
Mucin degradation	Negative	Negative	Positive	Negative

### 3.2 DW2009 toxicity study

#### 3.2.1 Subacute repeated-dose oral toxicity study

During the subacute toxicity study, no deaths or abnormal clinical signs were observed across all groups. In particular, no treatment-related changes in autonomic activity, movements, or behavior were noted in either sex at any dose level. Body weight and food consumption were comparable between the control and DW2009-treated groups throughout the study period (Supplementary Figure 1). The hematological results showed that platelet counts of female rats were lower in the low- and high-dose DW2009 groups than the control group, whereas no decrease was observed in males. Other hematological parameters showed no statistically significant differences between the control and DW2009-treated groups for either sex (Table 5). Serum biochemical parameters did not significantly differ between the control and DW2009-treated groups for either sex (Table 6).

**Table 5:** Hematological parameters of male and female rats treated with DW2009 for 28 days.

Parameter	Group (mg/kg bw/day)									*p*-value				
	Male				Female					Sex		Treatment		Interaction
	Negative control	DW2009 750 mg/kg	DW2009 1500 mg/kg	DW2009 3000 mg/kg		Negative control	DW2009 750 mg/kg	DW2009 1500 mg/kg	DW2009 3000 mg/kg					
White blood cell (×10^3^ cells/μL)	9.3 ± 0.9	10.87 ± 0.7	9.94 ± 0.6	8.88 ± 0.6		5.26 ± 0.9	5.83 ± 0.4	5.59 ± 0.5	5.98 ± 0.8	< 0.001		0.424		0.48
Red blood cell (×10^6^ cells/μL)	7.47 ± 0.2	7.53 ± 0.1	7.32 ± 0.2	7.41 ± 0.2		7.59 ± 0.1	7.46 ± 0.2	7.77 ± 0.2	7.51 ± 0.1	0.189		0.946		0.424
Hemoglobin (g/dL)	14.4 ± 0.2	14.7 ± 0.2	14.4 ± 0.4	14.3 ± 0.2		14.5 ± 0.1	14.5 ± 0.3	14.8 ± 0.3	14.8 ± 0.3	0.268		0.956		0.554
Hematocrit (%)	43 ± 0.7	44 ± 0.7	43.4 ± 1.0	43.1 ± 0.7		42.3 ± 0.4	42.4 ± 0.8	43.3 ± 0.8	42.8 ± 0.7	0.222		0.77		0.745
Platelet (×10^3^ cells/μL)	996 ± 27.7	1009 ± 41.6	1026 ± 53.7	959 ± 66.6		1022 ± 31.8	875 ± 25.9**	1018 ± 24.6	908 ± 30.4*	0.152		0.074		0.246
Mean corpuscular volume (fL)	57.7 ± 0.8	58.5 ± 0.5	59.4 ± 0.8	58.2 ± 0.6		55.8 ± 0.4	56.9 ± 0.6	55.9 ± 0.6	57.1 ± 0.8	< 0.001		0.439		0.318
Mean corpuscular hemoglobin (pg)	19.3 ± 0.3	19.5 ± 0.2	19.6 ± 0.3	19.4 ± 0.2		19.1 ± 0.2	19.4 ± 0.2	19 ± 0.2	19.7 ± 0.3	0.515	0.517		0.352	
Mean corpuscular hemoglobin concentration (g/dL)	33.5 ± 0.1	33.3 ± 0.2	33 ± 0.1	33.4 ± 0.2		34.3 ± 0.1	34.1 ± 0.1	34 ± 0.1	34.5 ± 0.1	< 0.001	0.015		0.463	

The data are presented as the mean ± SD (male/female n = 5).

*p < 0.05, **p < 0.01.vs. control group (Dunnett’s *t*-test)

**Table 6:** Serum biochemistry parameters of male and female rats treated with DW2009 for 28 days.

Parameter	Group (mg/kg bw/day)									*p*-value		
	Male				Female					Sex	Treatment	Interaction
	Negative control	DW2009 750 mg/kg	DW2009 1500 mg/kg	DW2009 3000 mg/kg		Negative control	DW2009 750 mg/kg	DW2009 1500 mg/kg	DW2009 3000 mg/kg			
Alanine aminotransferase(U/L)	28.6 ± 1.16	28.8 ± 2.55	29.1 ± 1.70	28.6 ± 1.70		24.6 ± 1.97	22.3 ± 0.98	23.7 ± 0.36	24.6 ± 1.43	< 0.001	0.901	0.853
Aspartate aminotransferase(U/L)	74.9 ± 0.80	80.6 ± 2.86	78.2 ± 1.79	71 ± 3.62		66.4 ± 1.57	65.2 ± 3.22	70.0 ± 4.70	72.1 ± 2.15	< 0.001	0.637	0.053
Alkaline phosphatase(U/L)	508 ± 52.32	630.5 ± 30.37	550.9 ± 35.15	534.2 ± 60.37		378.8 ± 48.34	458.1 ± 27.64	319.8 ± 13.37	399.3 ± 20.35	< 0.001	0.035	0.547
Glucose(mg/dL)	156 ± 18.78	148 ± 12.07	138 ± 6.26	141 ± 9.39		140 ± 3.13	145 ± 4.92	130 ± 6.71	132 ± 5.37	0.201	0.379	0.923
Blood urea nitrogen(mg/dL)	11.5 ± 0.58	12.1 ± 0.54	11.5 ± 0.40	13 ± 0.76		14.7 ± 1.03	13.9 ± 0.63	14.3 ± 1.25	13.3 ± 0.58	0.001	0.985	0.26
Creatinine(mg/dL)	0.38 ± 0.01	0.39 ± 0.01	0.39 ± 0.01	0.39 ± 0.01		0.45 ± 0.01	0.45 ± 0.01	0.44 ± 0.01	0.43 ± 0.01	< 0.001	0.973	0.579
Total cholesterol(mg/dL)	69 ± 8.50	67 ± 8.05	62 ± 6.71	71 ± 8.94		75 ± 3.13	94 ± 6.71	76 ± 5.81	88 ± 11.18	0.007	0.355	0.595
Triglycerides(mg/dL)	34 ± 5.37	37 ± 6.26	39 ± 4.02	41 ± 3.13		14 ± 3.13	14 ± 1.79	11 ± 1.79	13 ± 2.24	< 0.001	0.892	0.669
Total protein(g/dL)	5.5 ± 0.04	5.6 ± 0.09	5.4 ± 0.04	5.4 ± 0.09		5.8 ± 0.04	5.7 ± 0.09	5.7 ± 0.18	5.8 ± 0.13	< 0.001	0.363	0.698
Albumin(g/dL)	2.3 ± 0.04	2.4 ± 0.04	2.2 ± 0.04	2.3 ± 0.04		2.7 ± 0.04	2.6 ± 0.04	2.6 ± 0.04	2.7 ± 0.08	< 0.001	0.434	0.828
A/G ratio	0.73 ± 0.02	0.73 ± 0.02	0.72 ± 0.02	0.76 ± 0.04		0.85 ± 0.02	0.84 ± 0.02	0.86 ± 0.04	0.86 ± 0.04	< 0.001	0.799	0.952

The data are presented as the mean ± SD (male/female n = 5).

#### 3.2.2 Subchronic oral toxicity study

No mortality occurred and no signs of toxicity were noted in clinical observations including autonomic activity, movements, and behavior during the 90-day study. Body weight gain and food consumption remained comparable between the DW2009-treated and control groups (Supplementary Figure 2). Hematological analysis revealed elevated platelet counts exclusively in the low-dose female group as compared to the control (Table 7). Biochemical parameters showed increased blood urea nitrogen in mid- and high-dose male rats and gamma-glutamyl transferase across all female treatment groups. Notably, these alterations were not dose-dependent, indicating no direct correlation with exposure levels (Table 8). Urinalysis and histopathological evaluations revealed no treatment-related abnormalities (Table 9). There were no adverse findings. The no-observed-adverse-effect level (NOAEL) of DW2009 was 3000 mg/kg body weight/day.

**Table 7:** Hematological parameters of male and female rats treated with DW2009 for 90 days.

Parameter	Group (mg/kg bw/day)									*p*-value		
	Male					Female				Sex	Treatment	Interaction
	Negative control	DW2009 750 mg/kg	DW2009 1500 mg/kg		DW2009 3000 mg/kg	Negative control	DW2009 750 mg/kg	DW2009 1500 mg/kg	DW2009 3000 mg/kg			
White blood cell (×10^3^ cells/μL)	10.8 ± 2.25	9.80 ± 2.61	9.75 ± 3.00		10.14 ± 2.07	4.66 ± 1.43	4.10 ± 1.32	3.98 ± 1.27	4.78 ± 1.65	< 0.001	0.487	0.947
Red blood cell (×10^6^ cells/μL)	8.52 ± 0.28	8.38 ± 0.45	8.70 ± 0.42		8.49 ± 0.38	7.91 ± 0.40	7.76 ± 0.28	7.71 ± 0.27	7.92 ± 0.16	< 0.001	0.495	0.187
Hemoglobin (g/dL)	15.1 ± 0.5	14.9 ± 0.7	15.5 ± 0.9		15.3 ± 0.5	15.3 ± 0.5	14.9 ± 0.4	15.0 ± 0.7	15.0 ± 0.5	0.316	0.267	0.322
Hematocrit (%)	42.5 ± 1.5	41.9 ± 1.6	43.5 ± 2.1		42.8 ± 1.4	41.5 ± 1.7	40.8 ± 1.0	40.5 ± 1.6	41.2 ± 1.0	< 0.001	0.442	0.163
Platelet (×10^3^ cells/mL)	1007 ± 88	953 ± 107	984 ± 98		980 ± 111	865 ± 98	1003 ± 73^##^	911 ± 72	918 ± 169	0.019	0.63	0.047
Neutrophil (%)	16.4 ± 3.8	15.8 ± 6.6	17.5 ± 5.1		14.6 ± 6.1	14.0 ± 4.9	13.1 ± 3.6	16.6 ± 4.7	13.7 ± 2.9	0.111	0.246	0.896
Lymphocyte (%)	70.8 ± 5.3	72.0 ± 7.6	69.9 ± 5.7		73.2 ± 8.0	75.5 ± 5.7	77.2 ± 5.8	73.5 ± 4.9	76.1 ± 3.7	< 0.001	0.355	0.932
Monocyte (%)	11.1 ± 2.3	10.6 ± 2.0	10.9 ± 2.7		10.6 ± 1.8	8.5 ± 1.6	8.0 ± 2.5	8.1 ± 1.4	8.2 ± 1.7	< 0.001	0.873	0.989
Eosinophil (%)	1.4 ± 0.3	1.3 ± 0.3	1.4 ± 0.5		1.3 ± 0.5	1.8 ± 0.5.	1.6 ± 0.8	1.7 ± 0.8	1.7 ± 0.4	0.011	0.895	0.94
Basophil (%)	0.2 ± 0.1	0.3 ± 0.1	0.3 ± 0.1		0.3 ± 0.1	0.2 ± 0.1	0.2 ± 0.1	0.2 ± 0.1	0.2 ± 0.1	0.005	0.687	0.646
Reticulocyte (%)	3.03 ± 0.46	3.03 ± 0.31	2.93 ± 0.75		3.13 ± 0.45	2.86 ± 0.40	2.59 ± 0.57	2.88 ± 0.52	2.53 ± 0.53	0.008	0.823	0.334
Mean corpuscular volume (fL)	49.9 ± 0.41	50.0 ± 0.41	50.0 ± 0.41		50.4 ± 0.54	52.5 ± 1.5	52.6 ± 1.1	52.6 ± 0.5	52.1 ± 1.5	< 0.001	0.993	0.632
Mean corpuscular hemoglobin (pg)	17.8 ± 0.16	17.8 ± 0.16	17.8 ± 0.19		18.0 ± 0.16	19.3 ± 0.6	19.2 ± 0.5	19.4 ± 0.6	19.0 ± 0.7	< 0.001	0.932	0.314
Mean corpuscular hemoglobin concentration (g/dL)	35.6 ± 0.09	35.7 ± 0.09	35.6 ± 0.19	35.6 ± 0.09		36.9 ± 0.5	36.6 ± 0.3	36.9 ± 0.4	36.5 ± 0.4	< 0.001	0.296	0.123
Prothrombin time (s)	17.5 ± 0.7	17.7 ± 0.9	17.4 ± 1.1		18.0 ± 0.6	17.5 ± 0.7	17.6 ± 0.5	17.6 ± 0.7	17.6 ± 0.9	0.677	0.48	0.651
Activated partial thromboplastin time (s)	16.5 ± 1.5	16.1 ± 1.4	16.6 ± 0.9		15.6 ± 1.5	14.6 ± 1.4	15.4 ± 1.5	13.9 ± 1.5	15.1 ± 1.5	< 0.001	0.669	0.055

The data are presented as the mean ± SD (male/female n = 10).

#*p* < 0.05, ##*p* < 0.01 vs. control group (Steel’s test)

**Table 8:** Serum biochemistry parameters of male and female rats treated with DW2009 for 90 days.

Parameter	Group (mg/kg bw/day)									*p*-value		
	Male				Female					Sex	Treatment	Interaction
	Negative control	DW2009 750 mg/kg	DW2009 1500 mg/kg	DW2009 3000 mg/kg		Negative control	DW2009 750 mg/kg	DW2009 1500 mg/kg	DW2009 3000 mg/kg			
Sodium (mmol/L)	140.2 ± 1.2	140.4 ± 1.0	140.2 ± 1.0	140.4 ± 0.4		141.6 ± 1.6	141.6 ± 1.5	141.8 ± 1.4	141.1 ± 0.9	< 0.001	0.891	0.743
Potassium (mmol/L)	4.38 ± 0.28	4.29 ± 0.20	4.27 ± 0.32	4.23 ± 0.23		3.84 ± 0.28	3.99 ± 0.23	3.93 ± 0.27	3.82 ± 0.24	< 0.001	0.949	0.85
Chloride (mmol/L)	99.7 ± 1.4	99.6 ± 1.1	99.3 ± 1.0	99.7 ± 0.6		102.1 ± 1.6	101.9 ± 1.6	102.3 ± 1.3	101.2 ± 1.3	< 0.001	0.672	0.371
Alkaline phosphatase (U/L)	213.0 ± 37.1	215.1 ± 54.5	222.2 ± 46.6	228.3 ± 38.3		124.4 ± 27.3	114.3 ± 38.2	126.2 ± 37.1	120.3 ± 21.5	< 0.001	0.826	0.882
Aspartate aminotransferase (U/L)	81.3 ± 18.6	77.2 ± 14.3	76.4 ± 11.2	79.1 ± 12.3		82.3 ± 19.2	87.3 ± 18.7	101.4 ± 27.1	99.1 ± 15.6	< 0.001	0.384	0.155
Alanine aminotransferase (U/L)	38.9 ± 9.3	37.4 ± 7.9	35.5 ± 6.0	38.4 ± 3.5		30.5 ± 9.6	29.7 ± 7.7	31.6 ± 9.5	29.5 ± 4.8	< 0.001	0.958	0.713
Gamma glutamyl transpeptidase (U/L)	0.20 ± 0.09	0.19 ± 0.14	0.26 ± 0.09	0.22 ± 0.14		0.21 ± 0.08	0.47 ± 0.09^##^	0.36 ± 0.19^#^	0.42 ± 0.17^##^	< 0.001	0.011	0.008
Blood urea nitrogen (mg/dL)	13.2 ± 1.6	13.6 ± 1.7	15.5 ± 1.2**	15.2 ± 1.3*		16.5 ± 2.2	17.4 ± 1.9	17.9 ± 2.8	17.5 ± 2.8	< 0.001	0.023	0.65
Creatinine (mg/dL)	0.60 ± 0.05	0.59 ± 0.07	0.62 ± 0.02	0.59 ± 0.05		0.67 ± 0.07	0.70 ± 0.09	0.73 ± 0.09	0.65 ± 0.14	< 0.001	0.145	0.625
Total protein (g/dL)	5.6 ± 0.2	5.7 ± 0.2	5.8 ± 0.2	5.6 ± 0.2		6.2 ± 0.4	6.1 ± 0.4	6.0 ± 0.3	6.2 ± 0.3	< 0.001	0.996	0.108
Albumin (g/dL)	2.3 ± 0.1	2.3 ± 0.1	2.3 ± 0.1	2.2 ± 0.1		2.9 ± 0.2	2.7 ± 0.2	2.8 ±.2	2.9 ± 0.2	< 0.001	0.568	0.11
Triglycerides (mg/dL)	70 ± 27	66 ± 20	71 ± 28	47 ± 22		21 ± 6	19 ± 7	21 ± 10	22 ± 7	< 0.001	0.178	0.113
Total cholesterol (mg/dL)	66 ± 14	69 ± 19	84 ± 17	72 ± 9		77 ± 17	84 ± 20	78 ± 21	84 ± 12	0.033	0.325	0.216
Glucose (mg/dL)	147 ± 13	152 ± 21	149 ± 24	138 ± 15		133 ± 18	125 ± 11	123 ± 17	126 ± 15	< 0.001	0.468	0.379

The data are presented as the mean ± SD (male/female n = 10).

*p < 0.05, **p < 0.01 vs. control group (Dunnett’s test).

#*p* < 0.05, ##*p* < 0.01 vs. control group (Steel’s test).

**Table 9:** Urinary parameters of male and female rats treated with DW2009 for 90 days.

Parameter	Group (mg/kg bw/day)									*p*-value		
	Male					Female				Sex	Treatment	Interaction
	Negative control	DW2009	DW2009	DW2009	Negative control		DW2009	DW2009	DW2009			
		750 mg/kg	1500 mg/kg	3000 mg/kg			750 mg/kg	1500 mg/kg	3000 mg/kg			
pH	7.8 ± 0.42	7.9 ± 0.57	8.0 ± 0.00	7.9 ± 0.32	8.1 ± 0.42		8.2 ± 0.42	8.5 ± 0.53	8.6 ± 0.52	< 0.001	0.147	0.51
Specific gravity	1.03 ± 0.012	1.03 ± 0.010	1.04 ± 0.009	1.04 ± 0.012	1.03 ± 0.012		1.03 ± 0.012	1.04 ± 0.010	1.03 ± 0.012	0.773	0.179	0.117
Protein (mg/dL)	17.5 ± 12.08	20 ± 22.97	27.5 ± 18.45	17.5 ± 12.11	5.0 ± 10.54		0.0 ± 0.00	2.5 ±.7.91	15.0 ± 12.91	< 0.001	< 0.001	0.144
Ketone Body (mg/dL)	1.0 ± 2.11	3.0 ± 4.83	3.0 ± 2.58	5.0 ± 4.08	2.0 ± 2.58		0.5 ± 1.58	1.5 ± 2.42	5.5 ± 5.50	0.423	0.003	0.342
Bilirubin (mg/dL)	0.0 ± 0.00	0.1 ± 0.32	0.0 ± 0.00	0.0 ± 0.00	0.0 ± 0.00		0.0 ± 0.00	0.0 ± 0.00	0.0 ± 0.00	0.321	0.398	0.398
Occult Blood (erythrocytes/μL)	8.5 ± 16.67	6 ± 10.49	4.5 ± 8.32	5.5 ± 8.32	0.0 ± 0.00		0.0 ± 0.00	0.0 ± 0.00	0.0 ± 0.00	0.001	0.882	0.882

The data are presented as the mean ± SD (male/female n = 10).

### 3.3 Genotoxicity study

#### 3.3.1 Bacterial reverse mutation test

The Ames test was conducted using histidine-requiring *S. typhimurium* strains TA98, TA100, TA1535, and TA1537, as well as tryptophan-requiring *E. coli* strain WP2uvrA (pKM101). DW2009 did not influence the abundance of revertant colonies of any strain or at any of the tested concentrations, with or without metabolic activation. Conversely, all positive control groups exhibited a notable increase in revertant colonies following induction of the mutation (Table 10). Consequently, DW2009 showed no mutagenic activity up to the highest tested concentration.

**Table 10:** Ames test results of DW2009.

**DW2009 (μg/plate)**	**Number of revertant/plate**				
	**TA98**	**TA100**	**TA1535**	**TA1537**	**WP2uvrA** **(pKM101)**
+ S9					
312.5	33.0 ± 1.41	114.3 ± 9.05	11.2 ± 2.71	17.0 ± 1.67	117.3 ± 5.92
625	37.3 ± 4.50	99.8 ± 9.85	12.3 ± 2.66	17.3 ± 2.42	117.5 ± 6.09
1250	34.2 ± 1.17	112.7 ± 14.04	10.0 ± 1.26	17.0 ± 1.41	119.2 ± 4.26
2500	32.5 ± 1.38	107.3 ± 6.83	11.8 ± 1.17	17.5 ± 2.66	120.8 ± 4.62
5000	33.8 ± 2.04	119.0 ± 14.39	12.5 ± 1.05	15.7 ± 1.21	87.2 ± 37.43
Negative control	36.2 ± 0.75	102.5 ± 3.02	11.7 ± 1.03	17.8 ± 2.48	125.5 ± 4.97
Positive control	387.2 ± 4.02	969.5 ± 13.08	175.2 ± 3.06	233.8 ± 10.30	520.3 ± 5.75
- S9					
312.5	22.5 ± 5.01	98.7 ± 9.99	19.0 ± 1.55	10.8 ± 1.83	99.5 ± 7.26
625	25.7 ± 4.97	88.5 ± 17.07	16.2 ± 1.60	11.0 ± 3.35	92.8 ± 4.54
1250	22.0 ± 4.86	90.2 ± 8.59	16.2 ± 1.60	11.8 ± 1.33	91.2 ± 8.42
2500	21.8 ± 5.15	89.8 ± 8.61	16.5 ± 138	11.0 ± 0.89	85.2 ± 4.07
5000	22.2 ± 2.23	94.5 ± 11.00	16.2 ± 1.47	10.8 ± 1.17	84.2 ± 3.82
Negative control	24.5 ± 1.38	87.5 ± 8.41	15.5 ± 0.55	8.2 ± 1.33	95.5 ± 3.27
Positive control	725.3 ± 5.20	722.0 ± 3.35	576.5 ± 19.93	579.2 ± 11.55	715.5 ± 9.40

-S9, without metabolic activation; +S9, with metabolic activation.

The data are presented as the mean ± SD.

#### 3.3.2 Chromosomal aberration test

Along with S9 metabolic activation, short-term exposure (6 h) to DW2009 resulted in 0.0%–0.3% structurally aberrant cells, whereas the positive control (benzo[*a*]pyrene) yielded 18.7% aberrant cells. Without S9 activation, both short-term (6 h) and long-term (24 h) treatment with DW2009 resulted in 0.0%–0.3% aberrant cells. In contrast, the positive control (mitomycin C) yielded 17.0% and 29.5% aberrant cells following short- and long-term exposure, respectively. There was no statistically significant increase in the frequency of cells exhibiting chromosomal abnormalities compared with the negative control group under any of the tested conditions (Table 11).

**Table 11:** Chromosomal aberration test results of DW2009 in Chinese Hamster Lung (CHL/IU) cells.

**Test substance**	**Dose** **(μg/mL)**	**RPD** **(%)**	**Number of cells with structural aberration**									**Number of cells with numerical aberration**			**Others**
			**ctg**	**csg**	**ctb**	**cse**	**frg**	**gap**		**total (%)**		**end**	**pol**	**total (%)**	
								**ctg**	**csg**	**gap−**	**gap+**				
-S9, 6 h															
DW2009	1250†	84.2	0	0	0	0	0	0	0	0 (0.0)	0 (0.0)	0	1	1 (0.3)	0
	2500†	72.1	0	0	0	0	0	0	0	0 (0.0)	0 (0.0)	0	1	1 (0.3)	0
	5000†	66.8	0	0	0	0	0	0	0	0 (0.0)	0 (0.0)	0	0	1 (0.3)	0
Negative control	0	100	0.5	0	0.5	0	0	0	0	1 (0.3)	1 (0.3)	0	1	1 (0.3)	0
MMC	0.1	59.7	10.5	0	41	0.5	0	0.5	0	51** (17.0)	51.5 (17.2)	0	0	0 (0.0)	0
+S9, 6 h															
DW2009	625†	85.9	not observed												
	1250†	81.5	0	0	0	0	0	0	0	0 (0.0)	0 (0.0)	0	1	1 (0.3)	0
	2500†	75	0	0	0	0	0	0	1	0 (0.0)	1 (0.3)	1	0	1 (0.3)	0
	5000†	42.7	2	0	0	0	0	0	0	2 (0.7)	2 (0.7)	0	1	1 (0.3)	0
Negative control	0	100	0	0	1	0	0	0	0	1 (0.3)	1 (0.3)	0	1	1 (0.3)	0
B[*a*]P	20	52.1	10.5	0	47.5	0.5	0	0.5	0	56** (18.7)	56.5 (18.8)	0	0.5	0.5 (0.2)	0
-S9, 24 h															
DW2009	40.6†	81.7	not observed												
	81.3†	71.3	not observed												
	163†	66.7	0	0	0	0	0	1	0	0 (0.0)	1 (0.3)	0	1	1 (0.3)	0
	325†	57.8	0	0	0	0	0	0	0	0 (0.0)	0 (0.0)	0	0	0 (0.0)	0
	650†	43	1	0	0	0	0	0	0	1 (0.3)	1 (0.3)	0	0	0 (0.0)	0
Negative control	0	100	0	0	0.5	0.5	0	0	0	1 (0.3)	1 (0.3)	0	1	1 (0.3)	0
MMC	0.1	53.3	22.5	0.5	68.5	1	0	0	0	88.5** (29.5)	88.5 (29.5)	0	0	0 (0.0)	0

NC, Negative control; MMC, Mitomycin C; B[*a*]P, Benzo[*a*]pyrene; RPD, relative population doubling; ctg, chromatid gap; csg, chromosome gap; ctb, chromatid break; cte, chromatid exchange; csb, chromosome break; cse, chromosome exchange; frg, fragmentation; end, endoreduplication; pol, polyploidy; gap-, total number of cells with structural aberrations excluding gap; gap+, total number of cells with structural aberrations including gap.

**p < 0.01 vs. negative control (Fisher’s exact test), † precipitation

#### 3.3.3 Micronucleus test

The frequency of mnPCEs was significantly increased in mice treated with mitomycin C as compared to the negative control group. On the other hand, DW2009 did not increase the mnPCE frequency at any of the tested doses. The proportion of PCEs among total erythrocytes was comparable across all groups, indicating no evidence of bone marrow toxicity (Table 12). Furthermore, no mortality, clinical symptoms, or changes to body weight were observed in the DW2009-treated groups during the study period.

**Table 12:** *In vivo* micronucleus assay of *L. plantarum* C29 in male ICR mice.

Test substance	Dose (mg/kg)	PCE/RBC (%)	MNPCE/RBC (%)
DW2009	0	33.0 ± 0.71	0.035 ± 0.012
	1250	30.9 ± 0.36	0.035 ± 0.014
	2500	32.4 ± 0.67	0.050 ± 0.014
	5000	31.8 ± 1.07	0.055 ± 0.005
MMC	2	33.5 ± 1.16	6.255 ± 0.246**

PCE, polychromatic erythrocyte; MNPCE: micronucleated polychromatic erythrocyte; RBC, red blood cells; MMC, mitomycin C

**p < 0.01 vs. negative control (Mann–Whitney test)

## 4 Discussion

The present study provides comprehensive safety assessment data for *L. plantarum* C29 and DW2009 supporting the application of both in foods through *in vitro* and *in vivo* evaluations. The results show no evidence of any toxicological effects of *L. plantarum* C29 or DW2009.

Microbiological analysis confirmed that *L. plantarum* C29 fulfilled the EFSA antibiotic susceptibility criteria with all MIC values below established cut-offs for *Lactobacillus spp* [21]. Genomic analysis identified only one antimicrobial resistance gene (*vanY*), consistent with intrinsic vancomycin resistance commonly observed in *Lactobacilli* and not considered a safety concern [32], [33]. Hemolysis testing showed that *L. plantarum* C29 was γ-hemolytic, confirming a lack of hemolytic activity. The absence of cytotoxicity, virulence factors, toxin-encoding genes, enterotoxin and endotoxin production, BA production, and mucin-degrading activity further confirms the nonpathogenic characteristics of this strain.

*L. plantarum* C29 at 10^6^–10^7^ CFU produced basal levels of D-lactate comparable to the negative control. While D-lactate production was significantly increased at 10^8^ CFU, the concentration was lower than that of other *Lactobacillus* strains [34], [35]. In addition, the strain lacked BSH activity, which reduces the potential for interference with host bile metabolism. These results suggest that *L. plantarum* C29 has a favorable safety profile under standard administration regimens.

In the *in vivo* repeated dose toxicity studies, DW2009 was generally well tolerated. No DW2009-related mortality or adverse clinical signs were observed in both the 28-day subacute or 90-day subchronic oral toxicity studies. Although a prominent sex-related effect was detected for most of the parameters, this is attributable to the normal sexual dimorphism of rats rather than to any treatment. Platelet counts were decreased in females receiving DW2009 at doses of 750 and 3000 mg/kg/day in the 28-day study, but were increased only at the lower dose in the 90-day study. This inconsistency in the direction of change between studies indicates that the platelet findings reflect incidental variation rather than a treatment-related effect. In the 28-day study, a platelet count below the historical reference range was observed in one female rat at the 3000 mg/kg/day dose. In the 90-day study, similar decreases were noted in one female rat from the negative control group and in two female rats at the 3000 mg/kg/day dose. Because these changes were neither dose-dependent nor accompanied by consistent alterations in other erythroid parameters, they were considered incidental findings and unrelated to DW2009 administration. Two-way ANOVA revealed that mean corpuscular hemoglobin concentration and alkaline phosphatase showed sex and treatment effects but no interaction effects, and no concordant changes were observed in other mechanistically related analytes in the 28-day study. In the 90-day study, gamma-glutamyl transferase was elevated at all doses in females, but this was not accompanied by changes in alanine transaminase, aspartate transaminase, or alkaline phosphatase. Likewise, the increase in blood urea nitrogen in males at doses of 1500 and 3000 mg/kg/day was not mirrored by any changes in creatine, and all gamma-glutamyl transferase and blood urea nitrogen values remained within the normal range [36], [37]. In addition, the treatment effects observed in urinary protein and ketone were caused by interanimal variability and lacked any dose–response trend. The absence of associated histopathological correlations further support the tolerability of DW2009 at the administered doses. Taken together, these results established a NOAEL of 3000 mg/kg/day for DW2009, providing substantial safety margins for human use [38], [39]. Furthermore, genotoxicity studies, including the Ames test, chromosomal aberration test, and micronucleus test, demonstrated that DW2009 does not have mutagenic activity.

In a previous clinical study, seven adverse events were reported for the treatment group and five for the placebo group. However, this difference in the incidence rates was not statistically significant. One serious adverse event occurred during the study, but was not related to DW2009. Also, there were no clinically significant changes to the vital signs, body mass index, or laboratory findings between the treatment and placebo groups, indicating a favorable safety profile in humans [20].

Together, these findings provide consistent and robust evidence that DW2009 is safe for human consumption. The lack of microbiological and toxicological hazards, along with the absence of clinically relevant adverse effects in humans, supports its potential as a functional food ingredient.

## 5 Conclusion

Comprehensive safety assessment of DW2009, comprising *L. plantarum* C29 and fermented soybean powder, demonstrated that DW2009 exhibits a safety profile suitable for commercial application in foods and dietary supplements. Evaluation of *L. plantarum* C29 confirmed the absence of antibiotic resistance, hemolytic activity, cytotoxicity, BA production, BSH activity, mucin degradation, virulence traits, and toxin-related risks. No adverse effects were observed across any of the *in vitro* and *in vivo* assays conducted. Toxicological studies confirmed that DW2009 is well tolerated at high doses, establishing a NOAEL of 3000 mg/kg body weight/day in the 90-day toxicity study. Furthermore, the negative genotoxicity results and lack of treatment-related serious adverse events in a previous clinical study further substantiate the safety profile. Collectively, these findings support the safe consumption of DW2009 by the general population at intended use levels.

## CRediT authorship contribution statement

**Dong-Hyun Kim:** Writing – review & editing, Methodology. **Hyunju Kim:** Writing – review & editing, Writing – original draft, Software, Project administration, Conceptualization. **Daeyeon Won:** Supervision, Conceptualization. **Ji-Su Baek:** Methodology, Formal analysis.

## Ethical approval

The animal study protocol was approved by the Institutional Animal Care and Use Committee of Biotoxtech Co., Ltd. (IACUC approval numbers: 180082 for the 28-day toxicity study, 200395 for the 90-day toxicity study, and 180189 for the micronucleus test).

## Funding

The study was funded by DONGWHA Pharm Co., Ltd.

## Declaration of Competing Interest

The authors declare that they have no known competing financial interests or personal relationships that could have appeared to influence the work reported in this paper.

## Data Availability

Data will be made available on request.
