# Supplementary material for: Comprehensive safety evaluation of DW2009, a complex of Lactiplantibacillus C29 and fermented soybean powder
Source: Toxicol Rep. 2026 Jun 24;17:102299. doi: 10.1016/j.toxrep.2026.102299 (PMC13318553; doi:10.1016/j.toxrep.2026.102299)
Supplement: Supplementary file 1 — Supplementary material [file mmc1.docx]

***Supplementary Materials***

| Sample | Dilution | Agglutination result | Interpretation |
| --- | --- | --- | --- |
| *L. plantarum* C29  culture supernatant | 1:1 | – | Negative |
|  | 1:2 | – | Negative |
|  | 1:4 | – | Negative |
|  | 1:8 | – | Negative |
|  | 1:16 | – | Negative |
|  | 1:32 | – | Negative |
|  | 1:64 | – | Negative |
| Positive control  (*B. cereus* toxin) | 1:1 | +++ | Positive |
|  | 1:2 | ++ | Positive |
|  | 1:4 | ++ | Positive |
|  | 1:8 | ++ | Positive |
|  | 1:16 | ++ | Positive |
|  | 1:32 | + | Positive |
|  | 1:64 | – | Negative |

**Supplementary Table S1.** Detection of *B*. *cereus* enterotoxin in the culture supernatant of *L*. *plantarum* C29.

Agglutination intensity was scored as follows: −, none; +, weak; ++, moderate; +++, strong. The results are representative of three independent experiments.

**A B**

**C D**

**Supplemental Figure S1.** Body weight and food consumption of rats treated with DW2009 for 28 days.

Average body weight of (A) male and (B) female rats

Average food consumption of (C) male and (D) female rats

DW2009 low, 750 mg/kg body weight; DW2009 mid, 1,500 mg/kg body weight; DW2009 high, 3,000 mg/kg body weight

**A B**

**C D**

**Supplemental Figure S2.** Body weight and food consumption of rats treated with DW2009 for 90 days.

Average body weight of (A) male and (B) female rats

Average food consumption of (C) male and (D) female rats

DW2009 low; 750 mg/kg body weight, DW2009 mid; 1,500 mg/kg body weight, DW2009 high; 3,000 mg/kg body weight
